# The Sixteen Commandments of the Paris Academy of Medicine

**Published:** 1882-01-20

**Authors:** 


### THE SIXTEEN COMMANDMENTS OF THE PARIS ACADEMY OF MEDICINE.

TRANSLATED BY D. C. HOLLIDAY, NEW ORLEANS.

The Academy of Medicine in Paris has condensed into the following sixteen propositions the most important hygienic rules for the care and management of infants. We reproduce them here with the sincere hope that all mothers and nurses will commit them to memory and observe them as faithfully as the ten commandments of Holy Writ:

1. During the first year the only suitable nourishment for an infant is its own mother's milk, or that of a healthy wet nurse. Sucking should be repeated every two hours—less frequently at night.

2. When it is impossible to give breast milk, either from the mother or a suitable nurse, cow's or goat's milk given tepid, reduced at first one-half by the addition of water slightly sweetened, and after a few weeks one-fourth only, is the next best substitute.

3. In giving milk to an infant always use glass or earthen ware vessels, not metalic ones, and always observe the most scrupulous cleanliness in their management, rinsing whenever used. Always avoid the use of teats of cloth or sponge so frequently employed to appease hunger or quiet crying.

4. Avoid carefully all those nostrums and compounds so liberally advertised as superior to natural food.

5. Never forget that artificial nourishment, whether by nursing bottle or spoon (without the breast), increases to an alarming degree, the chances of producing sickness and death.

6. It is always dangerous to give an infant, especially during the first two months of its life, solid food of any kind—such as bread, cakes, meats, vegetables or fruit.

7. Only after the seventh month, and when the mother's milk is not sufficient to nourish the child, should broths be allowed. After the first year is ended then it is appropriate to give broth or paps made with milk and bread, dried flour, rice, and the farinaceous articles, to prepare for weaning. A child ought not to be weaned until it has cut its first 12 or 13 teeth, and then only when in perfect health.

8. A child should be washed and dressed every morning, before being nursed or fed. In bathing a child, temper the water to the weather, carefully cleanse the body, and especially the genital organs which require great cleanliness and care; and the head should be carefully freed from all scabs and crusts which may form. When the belly-band is used, it should be kept on for at least one month.

9. An infant's clothing should always be so arranged as to leave the limbs freedom of motion, and not to compress any portion of the body.

10. An infant's clothing should be studiously adapted to the weather; avoiding at all times, exposure to the injurious effects of sudden changes in temperature without proper covering; but nurseries and sleeping apartments should invariably be well ventilated.

11. An infant should not be taken into the open air before the fifteenth day after birth and then only in mild, fair weather.

12. It is objectionable to have an infant sleep in the same bed either with its mother or nurse.

13. No mother should be in too great a hurry to have a child walk; let it crawl and accustom itself to rising on its feet by climbing on articles of furniture, or assisted by the arms of a careful attendant. Great care should be taken in the too early use of baby-wagons, etc.

14. No trifling ailments in infants, such as colics, frequent vomiting, diarrhœa, coughs, etc., if persistent, should be neglected—a physician's advice should be at once obtained.

15. In cases of suspected pregnancy, either of mother or nurse, the child should be weaned at once.

16. A child ought to be vaccinated after the fifth month, or earlier should smallpox be prevalent.—[N. O. Med. and Surg, Jour.

